# Surgical Cleanliness

**Published:** 1899-04-22

**Authors:** 


### Surgical Cleanliness.

In a remarkable address delivered before the Gynæcological Society last month, Dr. Granville Bantock ran a tilt at modern bacteriology, maintaining that the relation of micro-organisms to disease was one of consequence rather than of cause, and that surgeons should trust to "simple cleanliness" instead of the elaborate "ritual" by which it is now usual to endeavour to obtain asepsis. In other words, that they should kick down the ladder by aid of which they have attained success. The position taken up by Dr. Bantock is one of entire disbelief in bacteria as causes of disease. He says: "I claim, then, to have shown that the poisons of variola, vaccinia, and syphilis are not, and cannot be, the product of a bacillus; that Loeffler's bacillus is not a constant, and therefore cannot be the essential, element for the production of an attack of diphtheria; that the essential element in the case of gonorrhœa is not the gonococcus; that the essential element in the case of typhoid fever is not the bacillus typhosus; . . . that there is no evidence worthy of the name that tuberculosis is due to the ravages of the tubercle bacillus," &c., all of which things "go to show that the modern doctrine of bacteriology is a gigantic mistake."

Nor does Dr. Bantock seem to hesitate to put into practice the extreme views which he so forcibly enunciates. He says: "I adopt none of the elaborate precautions of Dr. Howard Kelly, or the less complicated method described by Mr. Lockwood, beyond the simple washing of my hands previous to operation, and of my instruments after." And he asks, if the successes which he describes can be obtained by the adoption of "simple cleanliness in the common, everyday acceptation of the term," where is the necessity for the elaborate precautions advocated by the many surgeons who believe that germs are the cause of suppuration and other evils?

There can be no doubt that the occurrence of such cases as Dr. Bantock describes has always been a stumbling block to some, and the reading of such a paper as the one we refer to shows that the old feeling has not yet died away. It seems desirable then to point out that, although the examples brought forward are quite sufficient to show that germs do not have things all their own way; that some of the earlier and more extreme views as to the omnipresence of disease germs, and the teaching which sprung from those views, were erroneous; and also that we have much yet to learn as to the conditions which determine virulence in pathogenic micro-organisms; there is nothing in them to shake the position of modern bacteriology, or to militate in the slightest against the desirability of doing everything that is possible to obtain as complete an asepsis as is feasible in everything and everybody concerned in surgical work. That wounds will not heal unless complete asepsis is secured is no part of the creed of those who believe in antiseptics. The whole history of surgery shows that under all sorts of conditions wounds have healed well—sometimes; but bacteriology has shown how to turn that "sometimes" into an "always." Putting on one side, however, the mere argument from successes, which is pure empiricism, the weak point in the position taken up by those who, although maintaining the necessity for cleanliness, throw scorn upon the pathogenic properties of bacteria, is that they fail to show in what way dirt is injurious, or to explain what it is in cleanliness that renders it so essential to surgical success. To the surgeon who believes in germs these things are plain. He, even more than the simple-cleanliness-man, demands the most complete mechanical removal that is possible of all dirt, and he does so because he knows that in removing dirt he removes germs as well. But he also knows that by such means alone it is impossible to remove all germs, and the very same reason which teaches him to be clean teaches him also to strive after such a still further degree of asepsis as can only be attained by the use of heat and by the use of chemicals. If cleanliness does not mean germlessness it is a mere fetish; if it does, then every other means which helps towards the same goal is also good. But, indeed, it does not seem quite certain yet that those who deny the harmfulness of bacteria act quite up to the tenets of their creed, and it is fair to ask, as was asked when this paper was read, Why do they boil their ligature silk if not to kill the germs it may contain?

